# Correction: Protocol for research examination of individual suicides occurring in chronic pain: A qualitative approach to psychological autopsy methodology

**DOI:** 10.1371/journal.pone.0339797

**Published:** 2025-12-26

**Authors:** Stefan G. Kertesz, Allyson L. Varley, April E. Hoge, Thomas E. Joiner, Beth D. Darnall, Kate M. Nicholson, Lera A. Fuqua, Mary F. Gilmore, Kevin R. Riggs, Stephanie A. Gamble, Adam J. Gordon, Yogesh Dwivedi, Mark Flower, Christin Veasley, Carla Stumpf Patton, Ashley S. Leal, Dawn M. Gibson, James E. Elliott Jr, Megan B. McCullough

The 14th author’s name is spelled incorrectly. The correct name is: Christin Veasley.
