# Supplementary material for: Protocol for research examination of individual suicides occurring in chronic pain: A qualitative approach to psychological autopsy methodology
Source: PLoS One. 2025 Nov 14;20(11):e0329874. doi: 10.1371/journal.pone.0329874 (PMC12617874; doi:10.1371/journal.pone.0329874)
Supplement: S1 Appendix — (PDF) [file pone.0329874.s001.pdf]

CSI:OPIOIDS

Clinical Context of Suicide Following Opioid Transitions

UAB IRB Protocol #: IRB-300004342
VA IRBNet ID: 1681532
Principal Investigator Stefan G Kertesz, MD

The purpose of this survey is to learn about people who have died by suicide after a change in prescribed pain medicines. We believe it is critically important to learn about these very difficult situations, so that they can be prevented in the future. **For full details, click here.**

We are interested in finding people who are family members or partners of the person who died.
This survey presents a series of basic questions. If you proceed with the survey, you are offering consent to be in the survey. This is a research study, approved by the Central Institutional Review Board of the Department of Veterans Affairs, Veterans Health Administration and by the Institutional Review Board at the University of Alabama at Birmingham. Further information about who is involved in the study can be found at this link.

At the end of the survey, we will ask if you are willing to be contacted for a future study that would involve seeking an interview and medical records.

Also, if do not complete the survey at one sitting, we will send a reminder with information on how to complete the survey at a later time. This reminder would be by email or telephone, based on the preference you indicate in the survey.

Information obtained about you for this study will be kept confidential to the extent allowed by law. However, research information that identifies you may be shared with people or organizations for quality assurance or data analysis, or with those responsible for ensuring compliance with laws and regulations related to research.

If you provide responses suggesting that you may be at significant risk of harm to yourself or others, we will report it to a third party in the interest of protecting the rights and welfare of you or the persons at potential risk.

Participation in this survey is completely voluntary. There is no cost to participate in this survey. There is no compensation for participation in this survey.

If you have questions about your rights as a research participant, or concerns or complaints about the research, you may contact:

- VA Central IRB toll free at 1-877-254-3130
- UAB Office of the IRB (OIRB) at (205) 934-3789 or toll free at 1-855-860-3789. Regular hours for the OIRB are 8:00 a.m. to 5:00 p.m. CT, Monday through Friday.

The bottom of all pages of the survey will include crisis contact information including:
National Suicide Prevention Lifeline 24/7: Dial 988
Veterans Crisis Line 24/7: Dial 988 then Press 1

If your survey form skips questions, please notify the survey team at csiopioids@uabmc.edu and please begin the survey (if possible) on a different browser at https://go.uab.edu/csiopioids.
Please call our team at 866-283-7223, Option 1, if you need further assistance.

If you have questions about this study, you can contact the following members of the research team.

UAB Research and Recruitment Shared Facility: 1-866-283-7223, Option 1 *or* 205-975-7223.

Principle Investigator Dr. Stefan Kertesz: skertesz@uabmc.edu or 205-996-2866
Co-Investigator Dr. Allyson Varley: avarley@uab.edu or 205-996-2866
Project Coordinator Ms. April Hoge: april.hoge@va.gov or 205-578-2749

The bottom of all pages of the survey will include crisis contact information including:
National Suicide Prevention Lifeline 24/7: Dial 988
Veterans Crisis Line 24/7: Dial 988 then Press 1

If your survey form skips questions, please notify the survey team at csiopioids@uabmc.edu and please begin the survey (if possible) on a different browser at https://go.uab.edu/csiopioids. Please call our team at 866-283-7223, Option 1, if you need further assistance.

[New page]
Page 3

[Introductory text]: *This is a study about people who have died by suicide after a change in prescribed pain medicines. The following survey includes questions about suicide, which can be distressing for some people. Please know that your involvement is completely voluntary - you are able to stop and exit this survey at any time.*

We are interested in finding people who are family members or partners of the person who died.
We know that it can be hard to know for sure what happened. These questions can help us learn if our study team should contact you.

*Please note that we refer to "**opioids**".*

Opioids are strong pain medicines that require a doctor's prescription. They can be sold under many names. Sometimes people call them "narcotics". We will ask about specific medicines further below.

Q1: Do you believe that someone close to you died by suicide after a change in pain medication?

(-) Yes (if Yes continue)
(-) No (if No, continue to THANK-YOU SCREEN FOR DISQUALIFIED PERSONS, Page 10)

Q2: Are you age 19 or older?
(-) Yes (if yes continue)
(-) No (if No, continue to THANK-YOU SCREEN FOR DISQUALIFIED PERSONS, Page 10)

Q3: Do you reside in the United States of America?
(-) Yes (if yes continue)
(-) No (if No, continue to THANK-YOU SCREEN FOR DISQUALIFIED PERSONS, Page 10)

Q4: Did the person who died reside in the United States of America?
(-) Yes (if yes continue)
(-) No (if No, continue to THANK-YOU SCREEN FOR DISQUALIFIED PERSONS, Page 10)

RESPONSE LOGIC: If the answer is YES to ALL FOUR items, proceed to Q5.

If the answer to **any of the 4** items is NO, proceed to THANK-YOU SCREEN FOR DISQUALIFIED PERSONS

[New page]
Page 4

******If you begin the survey and do not finish, you are provided a code so that you can return within the next 2 weeks to complete the survey. If you provide an email address or phone number below, we will send a reminder with information on how to complete the survey at a later time. This reminder would be by email or telephone, based on the preference you indicate in the survey.*

Q5. Did this person ever serve in the US Armed Forces, including Army, Navy, Marines or Coast Guard, Air Force, Space Force, or National Guard?

(-) Yes, they were a member at the time of their death
(-) Yes, but they were no longer in the armed forces when they died
(-) No
(-) Don’t know/Not sure

Q6. What is your name?

First Name ________________________ [free text]
Last Name_______________________________

Q7. What is your E-mail address?

E-mail _______________________________

Q8. What is your preferred phone number?

( ) _____________________________

Q9. What state do you live in?

____

Q10. Would you prefer to do this survey online or do you prefer to be directly called by a member of the UAB research team?

(-) Online
(-) I request a telephone call for the survey

Would you like to participate in the CSI OPIOIDs Study?

(-) Yes, I agree to participate
(-) No, I do not agree to participate

RESPONSE LOGIC:

If YES, continue to Question 11
If NO, continue to a screen stating, “You have not consented to move forward with this survey. If you wish to take part in this survey, navigate back to the previous page, click “Yes, I agree to participate”, then click “Next Page” If you would like to complete survey

The bottom of all pages of the survey will include crisis contact information including:
National Suicide Prevention Lifeline 24/7: Dial 988
Veterans Crisis Line 24/7: Dial 988 then Press 1

If your survey form skips questions, please notify the survey team at csiopioids@uabmc.edu and please begin the survey (if possible) on a different browser at https://go.uab.edu/csiopioids. Please call our team at 866-283-7223, Option 1, if you need further assistance.

at another time, please return to https://go.uab.edu/csiopioids at any point in the future.”

Q11. What race best describes you?

(-) White
(-) Black or African American
(-) American Indian or Alaska Native
(-) Asian or Pacific Islander
(-) Other, please specify
(-) Decline to Answer ______________________

Q12. Do you consider yourself Hispanic/Latino/Latinx?

(-) Yes
(-) No
(-) Decline to Answer

Q13. What is your gender?

(-) Female
(-) Male
(-) Other_____________
(-) Decline to Answer

Q14. What city or town do you live in?

____

Q15. What is the best method to reach you?

(-) E-mail
(-) Telephone
(-) Postal mail
(-) Other Specify_________

Q16. Is there an alternate phone number you would like us to use?

(-) Yes if yes ( ) ____________
(-) No

Q17. What is your preferred mailing address? [request standardized fields including city and zip code]

Address ______________________
City__________________________
State___________
Zip ______________________

The bottom of all pages of the survey will include crisis contact information including:
National Suicide Prevention Lifeline 24/7: Dial 988
Veterans Crisis Line 24/7: Dial 988 then Press 1

If your survey form skips questions, please notify the survey team at csiopioids@uabmc.edu and please begin the survey (if possible) on a different browser at https://go.uab.edu/csiopioids. Please call our team at 866-283-7223, Option 1, if you need further assistance.

[New page]
Page 5

[Introductory text] ***Thank You, Now, these are questions about the person who died***

Q18. Who is the person that died (please provide the name of that person)
(-) First Name__________________
(-) Last Name __________________

Q19. Roughly how old was the person who died, when they died? [open ended text]
(-) Age______________

Q20. What was the gender of the person who died
(-) Female
(-) Male
(-) Other_____________

Please provide additional information about the gender of the person who died, if you wish [open ended text: limit 300 characters]

Q21. What was the race that best describes the person who died
(-) White
(-) Black or African American
(-) American Indian or Alaska Native
(-) Asian or Pacific Islander
(-) Other, please specify ______________________

Q22. Was the person who died Hispanic/Latino/Latinx
(-) Yes
(-) No

Q23. What was the marital status of the person who died
(-) Single, never married
(-) Married
(-) Widowed
(-) In a marriage-like relationship
(-) Divorced
(-) Separated

(-) Don't know/Not sure
(-) Other, please specify: ________________________

Q24. Where did the person live at the time of death? *(include city and state, if known) or indicate "I don't know")*
(-) City_________________
(-) State_________________
(-) Don't know/Not sure

Q25. What is your relationship to the person who died? [drop-down box]
(-) Spouse,
(-) Mother,
(-) Father,
(-) Sister,
(-) Brother,
(-) Son,
(-) Daughter,
(-) Other Specify______________ [Open-ended text]

Q26. Do you consider yourself to be in a family relationship with the person who died?
(-) Yes
(-) No

Q27. Did you live with the person who died at any time in the year before the death?
(-) Yes
(-) No

[New page]
Page 6
[Introductory text] *These are questions about the death itself.*

Q28. Please describe how the person died, to the best of your knowledge
Text field: limit 1500 characters

Q29. To the best of your recollection, what was the date on which this person died? (If you only know an approximate month or year, please provide that information.)
Text field: limit 500 characters

Q30. How confident are you that this death was an intentional act of suicide?

(-) 5= very confident
(-) 4=somewhat confident
(-) 3=neither confident nor unconfident
(-) 2=somewhat unconfident
(-) 1=very unconfident

Q31: To your knowledge did any of the following causes contribute to the death? Check all that apply [note: permit multiple checks on this one]

(-) 1= firearm/gun
(-) 2= suffocation/hanging
(-) 3= poisoning/overdose
(-) 4= medical illness
(-) 5= other (you may provide more information on the next question)
(-) 6= don't know/not sure

Q32. To your knowledge, was the death assessed to be a suicide by either a coroner, medical examiner or doctor?

(-) Yes
(-) No
(-) Don't know/Not sure

Q33. To your knowledge, in the last three years, did this person **<u>ever have an overdose</u>** where they needed to go to the emergency room or get medical care right away? Please respond "Yes" regardless of whether that event involved prescribed medicines or other substances.

(-) Yes
(-) No
(-) Don't know/Not sure

Q34. To your knowledge, was this person EVER told by a doctor or other health professional that he or she had (check ALL that apply)

(-)Depression
(-)Bipolar disorder
(-)An anxiety disorder like generalized anxiety or panic disorder
(-)Post-traumatic stress disorder (PTSD)
(-)A personality disorder, for example “borderline personality disorder”
(-)Schizophrenia
(-)Another psychological disorder
(-)None of these apply
(-)Don’t know/Not sure

Q35. Do you have a death certificate?

(-) Yes
(-) No

Q36. Was there an online obituary?

(-) Yes
(-) No
(-) Don’t know/Not sure

Q37: To your knowledge, how many times in the past, if any, did the person attempt to die by suicide?

[BLANK] times

[New page]

Page 8

These are questions about changes in pain medication. We understand you may feel uncertain regarding these questions. Please answer to the best of your ability.

Q38. To the best of your understanding, did the person who died ever have chronic pain lasting more than 3 months?

(-) Yes
(-) No
(-) Don't know/Not sure

****RRSF REQUEST: IF ANSWER TO Q38 IS "No" OR "Don't know/Not sure", SURVEY SKIPS TO Q43. IF ANSWER TO Q38 IS "Yes", CONTINUE SURVEY TO Q39.

Opioids are strong pain medications like hydrocodone, oxycodone, morphine, fentanyl, and brand-named drugs, and they require prescription from a doctor.

Q39. Was the person who died ever prescribed opioid medication for chronic pain?

(-) Yes
(-) No
(-) Don't know/Not sure

****RRSF REQUEST: IF ANSWER TO Q39 IS "No" OR "Don't know/Not sure", SURVEY SKIPS TO Q43. IF ANSWER TO Q39 IS "Yes", CONTINUE SURVEY TO Q40.

Q40. To the best of your knowledge, did the person who died experience any of the following changes in any opioid medication for chronic pain? (check all that apply; MULTIPLE RESPONSES ARE PERMITTED)?

(-) An opioid pain medication was reduced
(-) An opioid pain medication was stopped
(-) An opioid medication was increased
(-) An opioid medication was changed in some other way
(-) There was no change in any opioid medication
(-) Don't Know/Not Sure

Q41. Is there any more detail you would like to offer on how long the person who died received these medicines?

FREE Text

Q42. To the best of your knowledge, did the person who died describe distress about a change in pain medication? This may include distress before a change is made, or after. The distress might include anxiety, frustration, depression, or being upset.

(-) Yes
(-) No
(-) Don't Know/Not Sure

Q43. We encourage offering any additional information about what happened – even a sentence or two.

FREE text

**** RRSF REQUEST: MAKE THIS A REQUIRED QUESTION

Q44. To your knowledge, do you have legal authority to request medical records regarding the person who died, such as would be the case for a named "Executor of Estate"

(-) Yes
(-) No
(-) Don't know/Not sure

Page 9

THANK-YOU SCREEN FOR PRESUMPITVELY QUALIFIED PERSONS

We thank you for taking the time to complete this survey. Based on the information you have provided, we would like to contact you for a future study, when we obtain funding from an appropriate agency. Please provide any additional comments or questions in the text box below

Q45. Please provide any additional comments or questions in the text box below [Free text up to 2500 characters]

Q46. Are you willing to be contacted for a future study related to these deaths?

(-) Yes
(-) No

Page 10

THANK-YOU SCREEN FOR DISQUALIFIED PERSONS

The bottom of all pages of the survey will include crisis contact information including:
National Suicide Prevention Lifeline 24/7: Dial 988
Veterans Crisis Line 24/7: Dial 988 then Press 1

If your survey form skips questions, please notify the survey team at csiopioids@uabmc.edu and please begin the survey (if possible) on a different browser at https://go.uab.edu/csiopioids. Please call our team at 866-283-7223, Option 1, if you need further assistance.

"We're sorry. At this time, it does not appear that you meet the qualifications to complete this survey. Thank you for your time and interest in this research study."

Q47. May we contact you if there are future studies?

(-) Yes
(-) No
(-) Don't know/Not sure

If you have requested to be contacted for future studies, please provide the best method to reach you, whether that is email, telephone, or postal mail

Q48. What is the best method to reach you?

(-) E-mail
(-) Telephone
(-) Postal mail
(-) Other Specify__________

Q49. What is your E-mail address?

E-mail _______________________________

Q50. What is your preferred phone number?

( ) _____________________________

Q51. Is there an alternate phone number you would like us to use?

(-) Yes if yes ( ) _____________
(-) No

Q52. What is your preferred mailing address? [request standardized fields including city and zip code]

Address ________________________
City____________________________
State____________
Zip ________________________

Q53. Please tell us if there is another way you prefer to be reached (open text)
