# Supplementary material for: Protocol for research examination of individual suicides occurring in chronic pain: A qualitative approach to psychological autopsy methodology
Source: PLoS One. 2025 Nov 14;20(11):e0329874. doi: 10.1371/journal.pone.0329874 (PMC12617874; doi:10.1371/journal.pone.0329874)
Supplement: S2 Appendix — (PDF) [file pone.0329874.s002.pdf]

Step 1: *Contact Preference and Info*

i. Willing to be contacted
   a. IF **Q46 “Are you willing to be contacted for a future study related to these deaths?”** = Yes, THEN “Eligible”

ii. Must have provided some contact info (email, phone number)
   a. **Q7 “What is your email address?”** = [Text], THEN “Eligible”
   b. **Q8 “What is your preferred phone number?”** = [Text], THEN “Eligible”

Step 2: *Pass Prescreener*

i. Respondent must “believe” that “someone close” to them died by suicide “after a change in opioid medication”
   a. IF **Q1 “Do you believe that someone close to you died by suicide after a change in pain medication?”** = “Yes”, THEN “Pass”

ii. Respondent must be 19 or older
   a. IF **Q2 “Are you age 19 or older?”** = “Yes”, THEN “Pass”

iii. Respondent must live in the US
   a. IF **Q3 “Do you reside in the United States of America?”** = “Yes”, THEN “Pass”

iv. Decedent must have lived in the US
   a. IF **Q4 “Did the person who died reside in the United States of America?”** = “Yes”, THEN “Pass”

Step 3: *Note Participant/Decedent Relationship*

i. Remove “Self” reports
   1. If **Q25 “What is your relationship to the person who died?”** = “Other Specify” and **“Other specify, ___”** = “Self” or “Me” or “Myself”, etc. Take note and remove from eligible recruitment log.

ii. Note relationship proximity
   1. Included: **Family/Family-like** (Spouse, Immediate/Extended Family, SO/Partner)
   2. Included: **Friend** (unspecified or IRL/Ex-Spouse)
   3. Adjudicated: **Social media friend** but very knowledgeable (mention of durability of interaction, efforts to help the individual, received personal messages from individual, shared in online activity/campaign, etc.)
   4. Adjudicated: **Acquaintance** (Friend with only vague connection / lacking serious connection)
   5. Adjudicated: **Unspecified**. For people who select “Other” but do not specify. Look at all other qualitative responses to see if they explain their relationship.

Step 4: *Confidence of Suicide, Opioid Reduction/Stoppage, and Chronic Pain*

i. Confidence rating on suicide
   a. Category 1. High Confidence
      i. IF **Q25 “How confident are you that this death was an intentional act of suicide?”** = 5 (Very Confident)
   b. Category 2. Confident
      i. IF **Q25** = 4 (Somewhat Confident)

        ii. Review **Q28 "Please describe how the person died, to the best of your knowledge"** = **[**TEXT**]** AND THEN **Q45 "Please provide any additional comments in the text box below."** = [TEXT] for additional supporting data
    c. Category 3. Not Confident
        i. IF **Q25** = 1 or 2 or 3 (Unconfident, Somewhat Unconfident, Neither Confident nor Unconfident)
        ii. Review **Q28 "Please describe how the person died, to the best of your knowledge"** = **[**TEXT**]** AND THEN **Q45 "Please provide any additional comments in the text box below."** = [TEXT] for additional supporting data

ii. Confidence rating on opioid prescription
    a. Category 1. Easy Inclusion
        i. **Q40. "To the best of your knowledge, did the person who died experience any of the following changes in any opioid medication for chronic pain?"** IF Respondent selected **"Reduction"** AND/OR **"Stoppage",** AND no other answer option
    b. Category 2. Confusing Inclusion
        i. **Q40.** IF Respondent selected **"Reduction"** AND/OR **"Stoppage",** AND an additional answer option such as **"Increased", "Changed in some other way", "Don't know/Not Sure",** review **Q41 =** [TEXT] for additional supporting data
    c. Category 3. Ambiguous
        i. Based on review of these qualitative data, place individual in the following categories:
            1. Category 3a. Provisional Inclusion
                a. Although reduction and stoppage were not checked, the qualitative data reflects either a reduction/stoppage or a loss of access via prescriber or pharmacy
            2. Category 3b. Provisional Exclusion
                a. **Q40** IF Respondent selected **"Increase", "There was no change in opioid medication",** AND/OR **"Don't know/Not sure"**
                b. No new information supporting the idea that there was a reduction/stoppage (i.e. they got to the RedCap survey and provided nothing else to imply the decedent had an opioid reduction/stoppage)
            3. Category 3c. Middle Zone (ideally, we could follow-up with these if we have supplemental funding)
                a. Respondent is fairly sure that there was stoppage/threat of stoppage but there is no "hard evidence" of a reduction/stoppage
                b. Promise of more information after time has passed – we will follow-up with these individuals to give them info and state that we are interested in interviewing them if they feel they have enough info at this time

iii. Confidence rating on chronic pain experience
   a. Category 1. Easy Inclusion
      i. IF **Q38 "To the best of your understanding, did the person who died ever have chronic pain lasting more than 3 months?"** = "Yes"
   b. Category 2. Confusing Inclusion
      i. IF **Q38** = "Don't know/ Not sure"
   c. Category 3. Provisional Exclusion
      i. IF **Q38** = "No"
         a. No new information supporting the idea that there was chronic pain

Step 5: *Veteran Status*

i. Veteran status
   a. IF **Q5 "Did this person ever serve in the US Armed forces, including Army, Navy, Marines or Coast Guard, Air Force, Space Force, or National Guard?** = "Yes, they were a member at the time of their death" OR "Yes, but they were no longer in the armed forces when they died", THEN "Veteran"
   b. **Q5** = "No" or "Don't know/Not sure", THEN "Non-Veteran"

Step 6*: Legal Authority*

i. Ability to request medical record
   a. If **Q44 "To your knowledge, do you have legal authority to request medical records regarding the person who died, such as would be the case for a named "Executor of Estate"?** = "Yes", participant can assist with acquiring medical records
