# Supplementary material for: Protocol for research examination of individual suicides occurring in chronic pain: A qualitative approach to psychological autopsy methodology
Source: PLoS One. 2025 Nov 14;20(11):e0329874. doi: 10.1371/journal.pone.0329874 (PMC12617874; doi:10.1371/journal.pone.0329874)
Supplement: S3 Appendix — (PDF) [file pone.0329874.s003.pdf]

RRSF Recruitment and Retention Shared Facility

Suicide and Emotional Distress Protocols

Safety Protocol

IF YOU FEEL A PERSON IS IN *"GRAVE"* DANGER OF HURTING THEMSELVES:

(1) Say: "**I am concerned about you. Are you planning on hurting yourself?**"

a. If yes: "**Do you have a plan as to what you will do to hurt yourself?**" (A "yes" to these questions indicate that the participant is serious about harming themselves). "**Can you stay on the line with me as we contact the Crisis Line together?**"

- If the person stays on the line: Start a 3-way call with the Crisis Line (988) or Veterans Crisis Line (988 then dial 1) if they tell you that they are a Veteran.
- If the person disagrees: Explain that we are obligated to seek assistance on their behalf and let them know that you will be sharing information with emergency services for their area.
- If the person hangs up the phone, attempt 1 call back. If they do not answer, call 911 and give them all of the information you have for this individual (name, phone number, location if possible, and what the person said that made you feel they are in grave danger of hurting themselves.)

(2) Send relevant information to the VA Study Team via encrypted email to csiopioids@uabmc.edu (Email Template provided)

(3) Text a member of the VA Study Team – work your way through this list:

a. When Dr. Kertesz is in office, text Stefan Kertesz (205 612 7998)
b. When Dr. Kertesz is away, text April Hoge (256 609 8531)
c. When both Dr. Kertesz and April are away, text Mary Gilmore (205 586 8572)

- Text message: "*We have activated the imminent risk of harm response to a person using 911 or 988. We are sending all contact information regarding this to csiopioids@uabmc.edu.*"

(4) Document conversation in REDCap database.

IF THE PERSON CONFIDES *THOUGHTS* OF SUICIDE BUT DOES NOT HAVE AN ACUTE RISK OF SELF HARM

(1) Say: "**I am concerned about you. Are you planning on hurting yourself?**"

a. If no: "**I appreciate you sharing this with me. I am concerned about you. For this study, we always offer to connect participants with the 988 Crisis Line. Would you like to be connected with 988?"**

- If the participant agrees: start a 3-way call with the Crisis Line (if the person says they are a Veteran, connect with the Veterans Crisis Line by clicking "1").
- If the participant disagrees: Indicate to them that you'll inform the study team via email "Given your situation, I am going to let the study team know that we had this conversation."
- If the participant hangs up: attempt 1 call back. If they do not answer, move forward with notifying the VA study team.

(1) Send relevant information to the VA Study Team via encrypted email to csiopioids@uabmc.edu (Email Template provided)

(2) Text a member of the VA Study Team – work your way through this list:

- When Dr. Kertesz is in office, text Stefan Kertesz (205 612 7998)
- When Dr. Kertesz is away, text April Hoge (256 609 8531)
- When both Dr. Kertesz and April are away, text Mary Gilmore (205 586 8572)
  - Text message: "*We have activated the imminent risk of harm response to a person using 911 or 988. We are sending all contact information regarding this to csiopioids@uabmc.edu.*"

(3) Document conversation in REDCap database.

In Both Cases: NOTIFY VA STUDY TEAM IMMEDIATELY!

THE **UNIVERStTY OF ALABAMA AT BIRMINGHAM**

Knowledge that will change your world

Where there is uncertainty on how to move forward, RRSF will text the VA clinician on-call. This will usually be Dr. ________. If he is away/not available, notify Dr. _______ AND Study Coordinator ______. The clinician on-call can decide to either provide info to the VA study team for next steps or call the participant directly (though this is not required).

In the case that RRSF finds out the participant is a Veteran – include this info in notification email.

Emotional Distress Protocol

At signs of distress you should continue to **speak** calmly and in a gentle tone. Feel free to say, "it sounds like some of these questions are hard for you- is it okay to continue?"

Assure them that you are comfortable continuing if they are, that you appreciate their sharing these sensitive experiences with you. If they prefer to stop, let them know that is okay too- you can reschedule the interview or stop (i.e., withdraw them from the study).

Understand that detecting distress may be harder over the telephone (since you will not be able to see the person). Going slowly and allowing ample time to think and respond to questions generally helps you to keep tabs on the respondent's level of distress.

"*A KEY CONSIDERATION is that you should always discuss any instances of emotional distress on the patient's, caregiver's or YOUR part with the RRSF Study Supervisor.***

SPECIFIC PROCEDURES:

If you sense that someone is really struggling emotionally during the phone call:

(1) Stop the interview to ask, **"How are you doing right now?"** Note: it might be difficult to know how someone is feeling due to the survey questions requiring short, one-word responses. Distress might not be easy to read in their voices. The how-are-you-doing question gives them a chance to express how they feel. Some people might be very expressive, others won't open up.

(2) After the person has responded to the question, you can ask them, "**Would you like to continue with the interview, or would you prefer to stop?"**
   a) If they would like to continue, then check after another couple of minutes of questioning to make sure they are okay.
   b) If they would like to stop, then ask if they would like to continue at another time or if they prefer not to finish the interview (make sure they realize either choice is perfectly fine).

(3) If they needed to stop the interview/if you are concerned about their frame of mind: Express concern about how he/she is feeling with the following suggestions by saying, "It sounds like you are dealing with some difficult issues. Is there someone you can talk to about this?"
   a) If yes: "**Can you reach that person today to talk?**"
   b) If no: "**Would you like to speak with someone with the 988 Lifeline? They provide support for anything folks might be struggling with and can lend an ear to listen if you feel like talking. I**

can connect you with them or just give you the number (988).”

(4) Document conversation and surveyor's response in patient's research study file and report all concerns immediately to RRSF Study Supervisors.

(5) Send relevant information to the VA Study Team via encrypted email to csiopioids@uabmc.edu (Email Template provided)

NOTIFY VA STUDY TEAM IMMEDIATELY!
