# Supplementary material for: Protocol for research examination of individual suicides occurring in chronic pain: A qualitative approach to psychological autopsy methodology
Source: PLoS One. 2025 Nov 14;20(11):e0329874. doi: 10.1371/journal.pone.0329874 (PMC12617874; doi:10.1371/journal.pone.0329874)
Supplement: S4 Appendix — (PDF) [file pone.0329874.s004.pdf]

Info From Survey:

- Participant:
- Decedent:
- Relationship:
- Decedent Age:
- Location:
- Death Info:
- Opioid Info:
- Veteran Status:
- Other relevant information:
    -

Intro

Introduce Yourself and Notetaker [Notetaker should also explain that once the recording starts, they will not speak, they'll just follow along to take notes and help in this conversation*]*

Suggested Script:

Thank you for joining us in this conversation. How are you feeling today? The plan for this conversation is to ask questions about [DECEDENT'S NAME]. Is that something you're comfortable doing at this time?

As you might expect, I'll be asking you many questions about the circumstances surrounding [DECEDENT'S NAME] passing, which I know will be difficult. I want to emphasize that the purpose of this interview is not to place blame or responsibility. We are trying to gather factors that surrounded [DECEDENT'S NAME] passing.

We believe that what we learn from this can help improve care for others, and help policymakers understand just how important it will be to protect safety for people like the ones we have lost, going forward.

If you are unsure of the answer to a question, it's fine to say so. If there is a question you'd like to not answer, you won't have to explain. And if you would like to stop the interview at any time, you're free to do so. I will also do brief check-ins with you throughout the interview to ask how you're feeling. If I'm using any language that you're not comfortable with, please let me know – We're going to use the word "death" in this interview, but if you would be more comfortable with a word like "passing" please let me know.

In the history of this type of interview, there isn't any report of people describing that they feel suicidal after describing the loss of somebody else. But, I want to tell you about how we will respond to distress.

If you were to describe real distress where you need additional help – we will provide support and resources on how to get help. This is something that the informed consent document also mentioned (and national and local resources are included at the bottom of that document).

Also, if you were to say that there was an <u>imminent</u> likelihood of harming yourself – then we would be under obligation to seek support on your behalf as per study requirements. It's not our desire for this to happen and we have not heard of this happening before.

I'll be recording this interview for data collection purposes, but all data will be stored in a secure location with tightly controlled access. Is it okay if I begin the recording now? // Do you have any questions before I begin the recording?

--Begin recording—

Survivor information

1. Tell me a little about yourself.

Relationship to the decedent
(*Verify decedent identity matches REDCap Survey Decedent*)

2. What name should I refer to when talking about your loved one?
3. What can you tell about [name]?
   - *Probe*: What is your relationship to [the decedent] (i.e., parent, spouse, friend)?
   - *If there is a disagreement between REDCap Decedent and Interview Decedent, please probe to verify which person the participant would like to discuss. Document the resolution. Proceed with interview.*

Decedent Information

PCQ-Host survey questions

The next few questions have response options – I'll read those after each question

4. What race(s) did they consider themself? **(Select all that apply.)**

○ White
○ Black or African American
○ American Indian or Alaska Native
○ Asian or Pacific Islander
○ Other, please specify: ______________________________

5. Was [decedent] of Hispanic, Latina/Latino, or Spanish origin?

○ No
○ Yes

6. What is [decedent's] gender?

○ Male
○ Female
○ Another Gender

7. How old was [decedent]?

- ALSO ASK: What was [his/her] date of birth?

8. How hard was it for [decedent] to pay for the very basics like food and heating in the year before their death?

- ○ Extremely hard
- ○ Very hard
- ○ Somewhat hard
- ○ Slightly hard
- ○ Not at all hard

9. In the **year before [decedent's] death**, did they receive financial support of any kind, such as veteran's pension, social security, WIC, unemployment compensation, food stamps, rent, or fuel programs?

- ○ No
- ○ Yes

10. What was the highest level of school [decedent] completed?

- ○ Less than a high school degree and no GED
- ○ High school degree or GED
- ○ More than a high school degree or GED

11. Did X have children?

- ○ No
- ○ Yes

If yes, use following probes:

Were they present when X died?

12. Do you know if X served in the armed services of the US or another country?

- ○ No
- ○ Yes

Is yes,

12.a. Did they ever engage in care with the VA or a Veterans Hospital?

- ○ No
- ○ Yes

13. What was [decedents] employment status in the **year prior to their death**? If there are equal times for more than one category, select the **one** category which **best** represents your current situation. Also, if you are self-employed or you own your own business, please select "Full time" or "Part time" (whichever is appropriate for you).

○ Full time (35 or more hours per week)
○ Part time (less than 35 hours per week)
○ Unemployed (out of work but looking for work)
○ Unemployed (out of work but not looking for work)
○ Unemployed due to disability
○ Retired (and not employed)
○ Student (and not employed)
○ Other, please specify: ______________________

14. Was X known to be lesbian, gay, straight, bisexual, transgender, or questioning?

To start…..

15. To start, tell me a little bit about [DECEDENT NAME]' Death

Suicide

I'm going to ask you some questions about things that X may have said or done before they died (if necessary: you can skip any question you don't feel comfortable/ready to answer)

16. Can you tell me the date of X's death and where they died (City, State AND County)?

17. To your knowledge, did a coroner/medical examiner conduct an examination of any kind?
    - *If yes*, do you know the declared cause of death?

18. In your opinion why do you think X took his/her own life?

19. What was the method he/she used?

20. Do you have any thoughts on what might account for their using that method?

21. Do you think his/her suicide was related to a specific problem or event? *(Likely that people will offer up answers to many of our pain questions here)*

○ No
○ Yes

○ Other

If yes, use following probes:

Tell me more about the problem or event

When problems like these arose, how did X typically handle them?

22. Are you aware if X spoke with anyone about this problem or event?

○ No
○ Yes

If yes, use following probes:

Can you tell me who X usually spoke with?

23. Do you know if that person encouraged X to seek help?

○ No
○ Yes

If yes, use following probes:

Do you know who they encouraged X to seek help from?

Did X seek that help?

Do you know when?

Do you know where he/she sought help from?

Reviewer: please annotate if the "help seeking" was related to suicidal feelings/pain/etc.

24. Did X ever talk about or share information about suicide or ending their own life with you or others?

○ No
○ Yes

If yes, use following probes:

Tell me more about how they spoke/shared info about the of ending their life?

(Did they speak to someone or talk about this on social media?)

25. Do you know if X ever tried to take his/her life in the past?

○ No
○ Yes

If yes, use following probes:

Can you tell me more about when he/she tried to end his/her life and how it was attempted?

26. Did X ever talk to you or share information on social media about his/her thoughts of suicide or suicide attempts in the past?

○ No
○ Yes

If yes, use following probes:

When did he/she talk to you or share information on social media about these thoughts or attempts? (this can be open but "when" could mean a life phase, or a particular recency)

27. What can you tell us about X's mood before they died?

28. What about the last time you heard from them?
    - When was that?

29. Did X ever drink alcohol?

○ No
○ Yes

If yes, use following probes:

Do you know if X was drinking on the day of their death?

30. Do you know if X ever used drugs that were not prescribed by health care providers?

○ No
○ Yes

If yes, use following probes:

Do you know if X was using non-prescribed drugs on the day of their death? And/or Cannabis?

31. Did X ever tell you whether they felt they had any kind of addiction?

○ No
○ Yes

If yes,

What did they say?

Did they ever seek any treatment for that?

If yes or no,

Do you believe they had any kind of addiction?

Pain Care

32. Can you tell me where X received healthcare? Can you tell me more about the type of care X received?
    - Make sure to get general timelines – if they mention a particular doctor or healthcare system, we need a timeframe for requesting records (doesn't have to be exact - even a 5-year window is helpful)

33. Did X have issues with pain?
    - What were they?

34. For about how long had x suffered pain? (*NOTE TO INTERVIEWER: Probe to find a more specific time – "Few months", "Few years",)*

○ Less than 36 months
○ 3-5 years
○ Over 5 years

35. What was their diagnosis? Diagnoses?

36. Did X have any physical challenges such as mobility issues or old injuries?

○ No
○ Yes

If yes, use following probes:

Would you describe those issues or injuries?

Did they improve or worsen over time?

How was it in that period before they died?

37. Tell me what you know about X's experience getting care for their pain.

Probe: What kind of provider/doctor was providing care? (*Beware of "they" as a stand-in for the front desk staff)*

38. Do you know what kind of pain treatments X received?

○ No
○ Yes

If yes, use following probes:

Can you tell me about those treatments?

Example probes: medication? Physical therapy? Pain psychology/CBT? Chiropractor? Interventional treatments? Other non-pharmacological treatments?

<u>Note</u>: If someone makes a lot of "I think" or "I assume" statements – ask them why they think/assume these things.

39. Can you describe who was involved in delivering health care for the pain problems in the last year?
    - Were there particular health systems or clinics or offices where X received this care?

40. Were there any changes in the care X received for pain in the 6 months to a year before death? What were they?
    - Did any strike you as particularly important? (cue: listen for recency, or conflict, or crisis)

41. Can you tell us about medications for pain and what changes might have happened with medications? (cue: be prepared to ask if meds ever were helpful at all, or were changed).

42. Did this include pain medicines being reduced or stopped or suggestion to X about reducing or stopping them?

○ No
○ Yes

If yes, use following probes:

- What did X say about how these changes or potential changes were communicated to him/her?

43. If pain medicines were changed, how did X handle that – did they go to:

○ A new doctor
○ An addiction program
○ An emergency room
○ Another medication or drug source? (if so specify)

44. Did they appeal to an authority in the health care system/insurance carrier/wherever their doctor worked?

○ No
○ Yes

If yes, use following probes:

- Tell me more about that.
- Are there any other steps they took to help (himself/herself) that you want to mention?

45. To what extent did X seem to agree or disagree with his/her doctors or care providers about the plan of care for pain?

46. Did you notice anything to show you that X was experiencing stress or emotional strain related to pain care or changes in care? Did you hear anything from others?

(**Optional**) Do you know if X felt that their pain problem could be helped? In other words: did they feel that adequate treatment was available?

◯ No
◯ Yes

If no, use following probes:

Can you describe why they did not feel their pain problem could be helped or that treatment was unavailable?

47. Do you think that X felt there were any barriers to getting adequate pain treatment?

○ No
○ Yes

If yes, use following probes:

What were some of those barriers? Were there barriers related to geography or transportation?

Do you know if X had concerns about the financial cost of pain treatment or other supportive services?

Could you describe those concerns?

48. From your point of view, did the pain care plan fit with what X needed to do?

And how X lived?

And what she or he could manage?

49. Do you think X's death was related to their medication change?

○ No
○ Yes

If yes, use following probes:

Why do you think that?

50. Do you know if there were any policies about opioids at the organization X sought care at?

○ No
○ Yes

If yes, use following probes:

If so, please explain.

51. Do you recall X experiencing any challenges with getting medication at the pharmacy or with their insurance?

○ No

○ Yes

If yes, use following probes:

If so, please explain.

Mental Health

52. Did X ever talk to you about symptoms of anxiety, depression or other distress?

○ No
○ Yes

If yes, use following probes:

Could you describe the symptoms you spoke about?

No or Yes – invite the interviewee to tell us what they felt the decedent was anxious/depressed about?:

If you noticed any symptoms of anxiety or depression, what can you tell me about that?

53. Was X ever diagnosed or receiving treatment for a mental health problem?

○ No
○ Yes

If yes, use following probes:

Where did they receive this care/treatment?

What were their diagnoses you know about?

How long ago was their diagnosis or treatment for a mental health problem?

May skip:

54. If yes, Do you know if X felt their mental health problem could be helped?

○ No
○ Yes

If no, use following probes:

Can you describe why they did not feel their mental health problem could be helped?

(**Optional – Important if they were NOT getting help/treatment**)

Do you know if X was aware of mental health or other supportive services to receive help?

○ No
○ Yes

(**Optional – important if participant did not mention a community org in answer above**)

Do you know if X felt that help was available through community mental health agencies or support or faith-based organizations?

○ No
○ Yes

May skip:

55. Do you think that X felt there were any barriers to getting help?

○ No
○ Yes

If yes, use following probes:

What were some of those barriers?

Was X concerned about the attitudes or judgements of friends and family if they sought care for a mental health concern?

56. Do you know if X experienced trauma as a child?

○ No
○ Yes

If yes, use following probes:

How do you perceive that affected them?

57. Was there an upcoming event that X may have been concerned about?

○ No
○ Yes

If yes, use following probes:

What was that event?

58. Did they mention any problems with concentration or thinking?

○ No
○ Yes

If yes, use following probes:

Could you describe what they said about concentrating or thinking?

59. What about irritability in the sense that [X] was easily perturbed or upset?

○ No
○ Yes

If yes, use following probes:

Could you describe what they said about irritability?

60. What about problems with anger (which I realize may or may not be the same thing)?

○ No
○ Yes

If yes, use following probes:

How did they describe problems with anger?

Did you ever witness X behave aggressively?

61. Are you aware of any physical fights that X was involved in?

○ No
○ Yes

If yes, use following probes:

Could you describe those fights for me?

Physical Health

Now I am going to ask about X's physical health.

62. How would you describe x's general health?

○ Excellent
○ Very Good
○ Good
○ Fair
○ Poor

63. Did you know of X having any sleep problems?

○ No
○ Yes

If yes, use following probes:

How did X describe these issues?

64. Did X mention any problems with hearing or vision?

○ No
○ Yes

If yes, use following probes:

How did X describe the hearing or vision issues?

Social Environment

To help me understand X's life I would like to begin with some questions about their family, work, and social life.

65. Was X able to ask for, and receive help from others? (Note: different types of support might come into play here – social, financial, etc.)

○ No
○ Yes

If yes, use following probes:

Whom did he/she get help from?

66. Did X have regular contact with other family members or friends in the last year of their life?

○ No

○ Yes

If yes, use following probes:

Was it different for family vs friends?

If yes, use the following probes:

Do you know if X chose not to have contact with particular friends/family?

Do you know if they were aware of any of the problems X was suffering from?

Can you share with me what you think they may have been aware of?

67. Was X involved in any online communities?

○ No
○ Yes

If yes, use following probes:

Did they receive support from those online communities?

68. Did X speak with a clergy member about their pain/distress?

○ No
○ Yes

69. To the best of your understanding, did X share with you any concerns about being a burden to others?

70. Did X describe playing an important role for family and/or friends? (Note: Thwarted Belongingness)

Now I’m going to ask you some question about X’s living arrangements and employment.

71. Was X living in any of the following places at the time of their death:

○ House
○ Apartment
○ Condo
○ Other (please specify)
○ No

72. If not, were they living in any of these places within the 3 years leading up to their death?

○ Shelter
○ friend/family member's homes
○ Car
○ Any other place not meant for sleeping (please specify)

73. Were they living with anyone?

○ No
○ Yes

If yes, can you tell me a little bit more about that? Tell me more about their relationship (how long had they been living together? If they were married, for how long were they married? Etc.)

74. Were there frequent changes in living arrangements in the year before X's death?

○ No
○ Yes

75. If not already covered: Was there a living situation change within the 30 days leading up to X's death?

○ No
○ Yes

If yes: Do you believe this might have been relevant to X's death?

○ No
○ Yes

76. Do you believe anything related to employment, or the receipt or non-receipt of benefits might have been relevant to X's death?

○ No
○ Yes

If yes, were there frequent changes in employment in the year leading up to their death?

○ No
○ Yes

If yes, use following probes:

Had their employment situation changed in the last 30 days?

Please describe how their employment changed.

77. Is there anything else you would like to share with me about X's family or social habits?

Legal issues

78. Did X face any legal issues within the year before their death?

○ No
○ Yes

If yes, were they related to:

○ Divorce or relationship
○ Alcohol or drug use
○ Fighting or violence
○ Being a victim of a crime (probe: violent or non-violent)
○ Engaging in a crime (probe: violent or non-violent)
○ Other (specify)
○ None

Firearm ownership and usage

79. Did X own a firearm?

○ No
○ Yes

If yes, use following probes:

Why do you think X owned a firearm?

Were they a hunter?

Did they have a license to hunt?

Were the guns kept locked up at home?

How were they kept locked?

Do you know if X ever attend a firearm safety course?

For Veterans

[ONLY ASK IF THEY RESPONDED “Yes” TO BEING A VETERAN]

80. What branch of service did they service in? Check all that apply.

○ Army
○ Navy
○ Air Force

○ Marines
○ Coast Guard
○ Don’t know

81. Do you believe their military service might have been relevant to X’s death? [Yes/No]

If yes, use following probes:

Do you know when X separated from the military?

When was that?

In your opinion, did X have a difficult time transitioning out of active service?

Can you describe your impression of that difficulty for me?

82. Do you know if X ever contacted the Veteran’s crisis line?

○ No
○ Yes

If yes, use following probes:

Tell me what you know about their experience with the crisis line?

FOR VETERANS WITH PAST VA CARE EXPERIENCE

[ONLY ASK IF THEY RESPOND “Yes” TO EVER BEING ENGAGED IN VA CARE]

83. Do you know if X felt that help was available through the Department of Veterans Affairs?

Probes: *for their pain? For suicide?*

84. Do you know if X had concerns about going to the VA for care?

○ No
○ Yes

If yes, use following probes:

Could you describe the concerns they had?

Close-Ended Questions

Thank you so much for answering these questions for us.

Before we finish the interview,

85. On the online survey you completed, you said you did/did not have legal authority to request X's medical record. Is this still true?

    If no:

    Do you know who might have legal authority to request X's medical record?

    Are you in communication with them/comfortable reaching out to see if they would be interested in helping to get X's records? *(Reassure respondent that we **will not** reach out to this individual)*

86. Is there anything else you want to share with us about the death of X?

Lastly, I have two questions about your opinion on how we can prevent future deaths like X's.

87. Are there any improvements that you think health care providers or health care systems could make to improve suicide prevention programs?

    What would those program improvements be?

88. What do you feel is the biggest barrier to preventing suicide in our country?

END RECORDING – Closing

- *We know these conversations can be difficult – We've included resources on the last page of your consent form. Please let me know if I can resend that info to you.*
- *We will be sending you a check-in email within the next week just to say hello and ask how you're feeling – would you be okay with that?*
- *Also, if there is anyone else who you think might have insight about what happened and you think might be open to talking with us, I'll include info like our study website in the email I send to you in a couple weeks.*
- *Medical Records:*
    - *(If already completed Medical Record process):*
    - *(If they haven't completed the Medical Record process): [restate their answer to Question 86].*
        - *If they do have authority: let them know we'll be sending them a form via postal mail for them to sign along with a request for a few additional forms like a copy of the death certificate and a copy of Executor of Estate forms)*
        - *If they don't have authority: Either ask them if they'd be comfortable reaching out to whoever has authority or ask if they would be open to signing the release form themselves – it's possible*
    - *If we will be mailing a record release form to them:*
        - *In the online survey you completed, said you **did/didn't** have a copy of the death certificate. Is that still true? If you're able, we'll request that you send a copy of*

the death certificate back with your signed medical record release form. We'll be mailing that to you soon.

- *If they do have the ability to request*:
  - **--What was [decedent]'s date of birth?**

- *Gift Cards:*
  - *Info **required** for securing their gift cards (we cannot override these requirements):*
    - ***Participant's First and Last name**:*
    - ***Mailing Address** (If they provided one in their screener survey, paste it here and verify with the participant that it's still correct):*
    - ***Date of Birth**:*
    - ***Phone Number**:*

Additional Thoughts from Interviewer/Notetaker:

- ***Interviewer's global assessment of knowledge of interviewee (On a Scale of 1-5):***
  - ***On a Scale of 1 to 5, where 1 is "Not very informative" and 5 is "Extremely informative"***
  - ***Interviewer/Notetaker's Score:***
